# Supplementary material for: A Nature-Inspired Nrf2 Activator Protects Retinal Explants from Oxidative Stress and Neurodegeneration
Source: Antioxidants (Basel). 2021 Aug 16;10(8):1296. doi: 10.3390/antiox10081296 (PMC8389314; doi:10.3390/antiox10081296)
Supplement: Supplementary file 1 [file antioxidants-10-01296-s001.zip › antioxidants-1312448-supplementary.pdf]

Supplementary figures

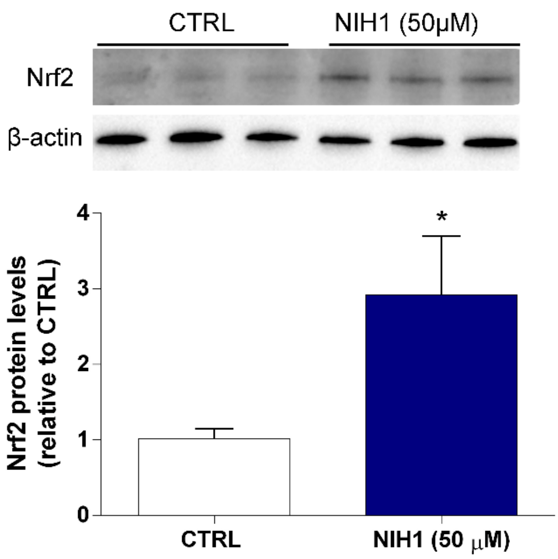

Figure S1. Western blot analysis of cytosolic protein fraction showing representative immunoreactive bands and quantitative densitometric analysis of the Nrf2 protein levels in CTRL explants and in explants treated with 50 μM NIH1 for 24 h. Mean ± SEM, unpaired t-test, n=3. *$p<0.05$ vs CTRL.

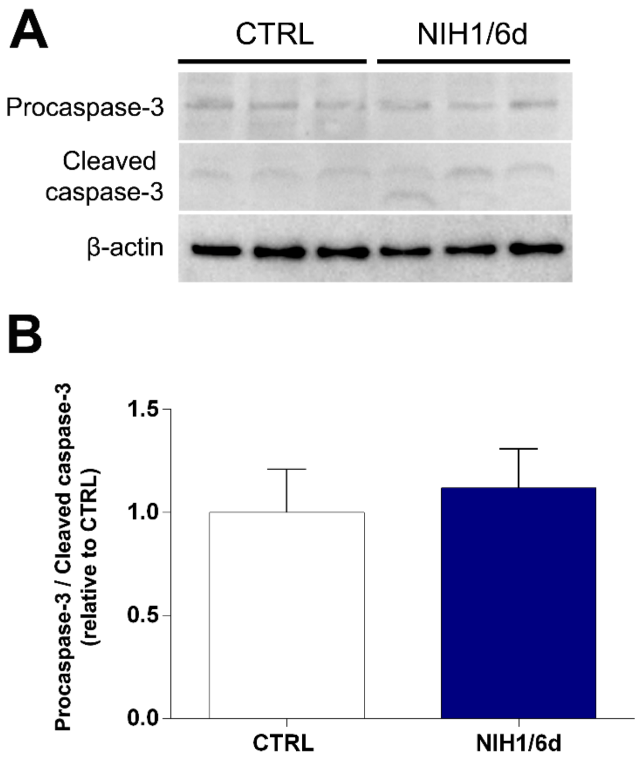

Figure S2. Effect of NIH1 on cleaved caspase-3/procaspase-3 ratio. Western blot of total protein fraction obtained with antibody Cell Signaling #9661, which recognizes both procaspase-3 and cleaved caspase-3, showing representative immunoreactive bands of cleaved caspase-3 and procaspase-3 (A) together with their ratio (B) calculated from the quantitative densitometric analysis of the respective immunoreactive bands, in CTRL explants and in retinal explants treated with 50 μM NIH1 for six days. Unpaired t-test, n=3.

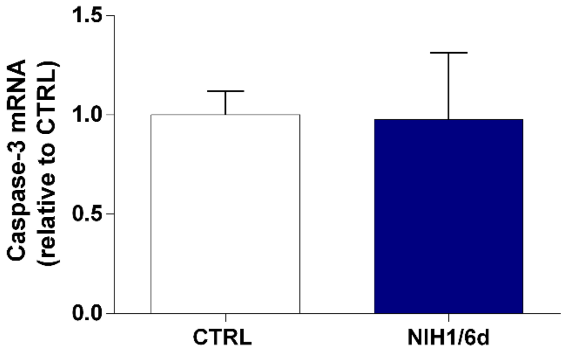

Figure S3. Effect of NIH1 on caspase-3 mRNA expression. qPCR analysis showing the relative levels of caspase-3 mRNA in retinal explants treated with 50 μM NIH1 for six days (NIH1/6d). CTRL, control, untreated explants. Mean ± SEM, unpaired t-test, n=3.

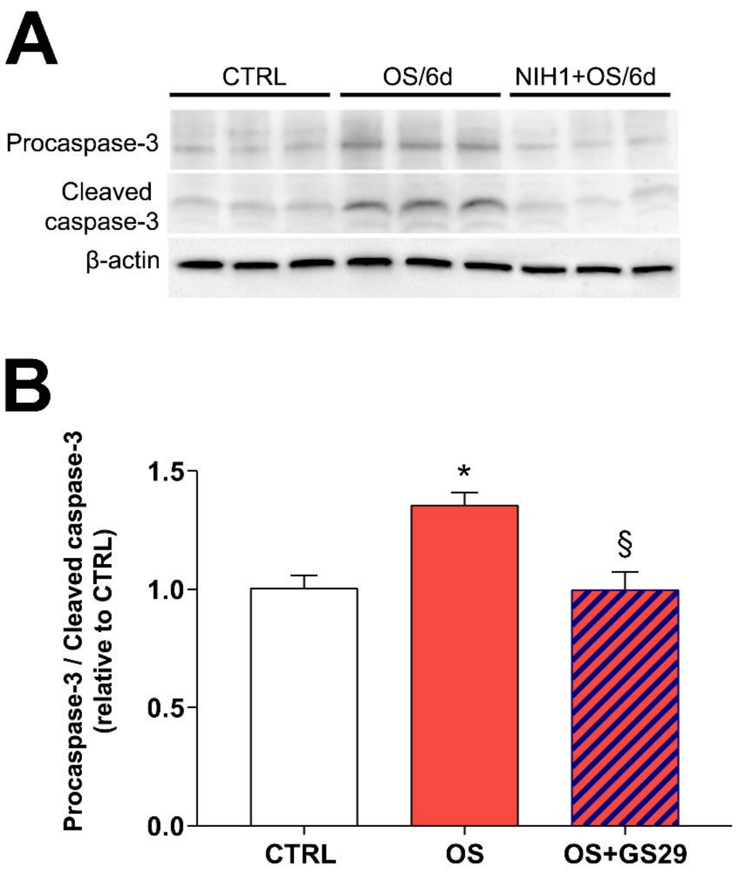

Figure S4. Effect of NIH1 on cleaved caspase-3/procaspase-3 ratio. Western blot of total protein fraction obtained with antibody Cell Signaling #9661, which recognizes both procaspase-3 and cleaved caspase-3, showing representative immunoreactive bands of cleaved caspase-3 and caspase-3 (A) together with their ratio (B) calculated from the quantitative densitometric analysis of the respective immunoreactive bands, in CTRL, OS/6d and NIH1+OS/6d explants. One-way ANOVA, n=3; *$p < 0.05$ vs CTRL; §$p < 0.05$ vs OS/6d.

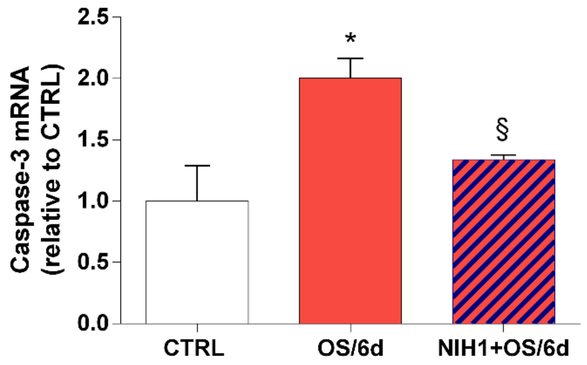

Figure S5. Effect of NIH1 on caspase-3 mRNA expression. qPCR analysis showing the relative levels of caspase-3 mRNA in control retinal explants (CTRL), in retinal explants incubated with 100 µM H_2O_2 for six days (OS/6d), and in retinal explants incubated with 100 µM H_2O_2 together with 50 µM NIH1 for six days (NIH1+OS/6d). Mean ± SEM, one-way ANOVA, n=3; *$p < 0.05$ vs CTRL; §$p < 0.05$ vs OS/6d.
